# Circumventing senescence is associated with stem cell properties and metformin sensitivity

**DOI:** 10.1111/acel.12889

**Published:** 2019-01-06

**Authors:** Xavier Deschênes‐Simard, Maxime Parisotto, Marie‐Camille Rowell, Benjamin Le Calvé, Sebastian Igelmann, Karine Moineau‐Vallée, Emmanuelle Saint‐Germain, Paloma Kalegari, Véronique Bourdeau, Filippos Kottakis, Nabeel Bardeesy, Gerardo Ferbeyre

**Affiliations:** ^1^ Department of Biochemistry and Molecular Medicine and CR‐CHUM Université de Montréal Montréal Québec Canada; ^2^ Cellular Biology Research Unit (URBC)‐NARILIS University of Namur Namur Belgium; ^3^ Massachusetts General Hospital Cancer Center Harvard Medical School Boston Massachusetts; ^4^ Present address: CHUM Montréal Québec Canada

## Abstract

Most cancers arise in old individuals, which also accumulate senescent cells. Cellular senescence can be experimentally induced by expression of oncogenes or telomere shortening during serial passage in culture. In vivo, precursor lesions of several cancer types accumulate senescent cells, which are thought to represent a barrier to malignant progression and a response to the aberrant activation of growth signaling pathways by oncogenes (oncogene toxicity). Here, we sought to define gene expression changes associated with cells that bypass senescence induced by oncogenic *RAS*. In the context of pancreatic ductal adenocarcinoma (PDAC), oncogenic KRAS induces benign pancreatic intraepithelial neoplasias (PanINs), which exhibit features of oncogene‐induced senescence. We found that the bypass of senescence in PanINs leads to malignant PDAC cells characterized by gene signatures of epithelial‐mesenchymal transition, stem cells, and mitochondria. Stem cell properties were similarly acquired in PanIN cells treated with LPS, and in primary fibroblasts and mammary epithelial cells that bypassed Ras‐induced senescence after reduction of ERK signaling. Intriguingly, maintenance of cells that circumvented senescence and acquired stem cell properties was blocked by metformin, an inhibitor of complex I of the electron transport chain or depletion of STAT3, a protein required for mitochondrial functions and stemness. Thus, our studies link bypass of senescence in premalignant lesions to loss of differentiation, acquisition of stemness features, and increased reliance on mitochondrial functions.

## 1 INTRODUCTION

A variety of oncogenes induce DNA damage, protein degradation, and mitochondrial dysfunction ultimately triggering a stable cell cycle arrest known as oncogene‐induced senescence (OIS). OIS is considered a powerful anti‐cancer response mediated by *bona fide* tumor suppressors such as p53, RB, and p16INK4a (Salama, Sadaie, Hoare, & Narita, 2014). In mice, expression of oncogenes in a variety of tissues leads to premalignant lesions with the characteristics of OIS that often progress to form malignant tumors (Collado & Serrano, 2010). Although inactivation of tumor suppressors such as p53 accelerates the formation of tumors in these mouse models (Collado & Serrano, 2010), the mechanism of senescence bypass in tumors that spontaneously arise from premalignant lesions remains unclear. In particular, there is an extensive reprogramming of the cancer genome resulting in loss of genetic programs of cell differentiation and gain of gene expression programs of embryonic stem cells (ESCs) (Ben‐Porath et al., 2008). While many comparisons have been made between tumor cells and their normal counterparts, there is much to learn by comparing malignant cells to their precursor premalignant lesions being both populations expressing the same driving oncogene.

To investigate the molecular changes associated with the transition from premalignant senescent lesions to malignant tumors, we took advantage of genetically engineered mouse models (GEMMs) of pancreatic ductal adenocarcinoma (PDAC) that mimic the progression of the human disease. Activating Kras mutations in the pancreas lead to premalignant lesions known as pancreatic intraepithelial neoplasias (PanINs), which are largely nonproliferative and contain cells with markers of cellular senescence (Caldwell et al., 2012). We thus compared the transcriptome and biological properties of PanIN and PDAC cells. PDAC cells express genes regulated by Stat3 and Myc and have low levels of genes repressed by NF‐κB. They also expressed mitochondrial genes and genes in common with stem cells. Consistent with their transcriptome, PDAC cells exhibited stem cell properties and displayed sensitivity to treatment with the mitochondrial complex I inhibitor metformin or to shRNAs against Stat3. Stemness was also stimulated in PanIN cells by LPS and in human primary cells that bypassed Ras‐induced senescence due to attenuation of ERK signaling. Taken together, our results link bypass of senescence with Stat3‐dependent stemness and metformin sensitivity and provide insights into the association between cancer and aging.

## 2 RESULTS

### 2.1 The transition from PanIN to PDAC involves acquisition of stem cell and epithelial‐mesenchymal transition gene expression modules

**Figure 1:**
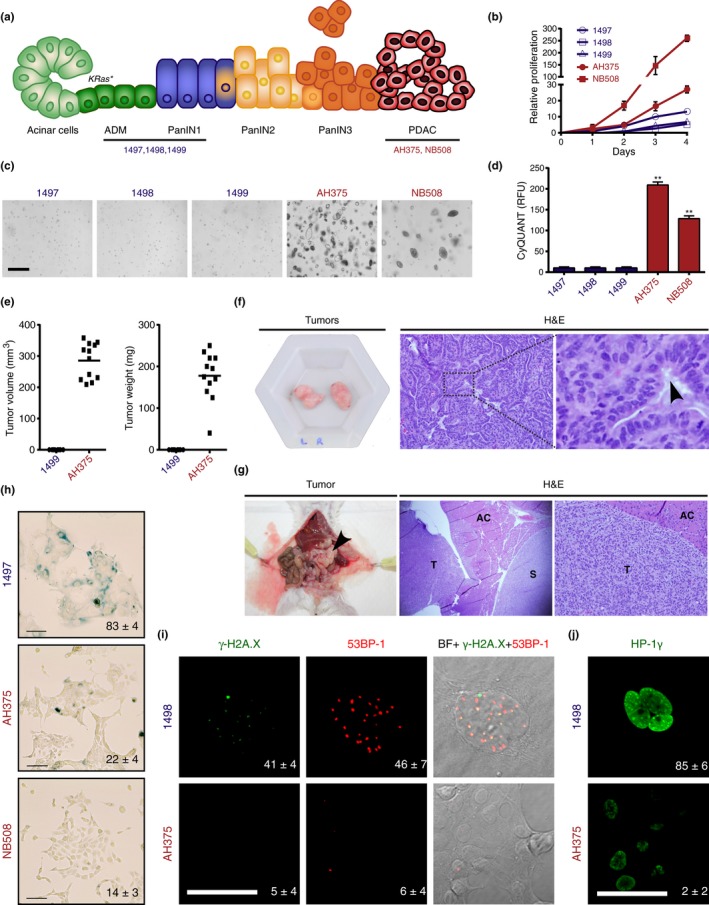
Establishment of an in vitro model of pancreatic cancer progression. (a) Illustration of stages in pancreatic cancer progression after oncogenic *ras* activation (Kras^*^) in vivo. Adapted from Wilentz et al. (2000). Mouse pancreatic ductal cell lines were established from the indicated lesions of *Pdx1‐Cre;LSL‐Kras^G12D^*mice. (b) Proliferation of the indicated mouse cell lines measured by MTT. The relative proliferation represents the fold of OD at 500 nm over the indicated period of time. Each point represents the mean of triplicates ± *SD*. (c) Cell lines from pancreatic ductal adenocarcinoma (PDAC) form colonies in soft agar, but not cell lines from ADM/PanIN1 lesions. Scale bar = 400 μm. (d) Quantification of proliferation in soft agar over a period of 7 days for the indicated cell lines. Results were obtained using the CyQuant GR dye and are expressed as relative fluorescence unit (RFU) at 520 nm. Mean of triplicates ± *SD*, **p < 0.01. (e) Tumor volume and weight 15 days after subcutaneous injection of 5 × 10^5^ 1,499 or AH375 cells into SCID mice. Only AH375 cells form tumors. (f) Phenotype and histology of subcutaneous tumors formed by AH375 cells. H&E, hematoxylin and eosin. Black arrow, ductal histology (g) Phenotype and histology of tumors formed following orthotopic injection of AH375 cells into the pancreas of SCID mice. H&E, hematoxylin and eosin; AC, normal acinar cells; S, spleen; T, tumor. (h) Percentage of SA‐β‐Gal‐positive cells in the indicated cell lines. The average and *SD* of triplicates of 100 cells counts are indicated at the bottom of each panel, *n* = 3. Scale bar = 10 μm. (i) Indirect immunofluorescence staining with anti‐γ‐H2AX and anti‐p53BP1 antibodies of PDAC cell line AH375 and PanIN cell line 1,498, scale bar = 10 μm. The percentage of cells with more than five foci is indicated at the bottom right of each panel. (j) Indirect immunofluorescence staining with anti‐HP1γ antibody of PDAC cell line AH375 and PanIN cell line 1,498, scale bar = 10 μm. The percentage of cells with more than five foci is indicated at the bottom right of each panel. We counted 50 cells three times in two independent experiments

Low‐grade PanIN lesions (PanIN1) are frequent in old individuals without pancreatic cancer but high‐grade lesions (PanIN2 and PanIN3) are rare in the normal pancreas. In contrast, PanIN3 lesions are frequent in patients with pancreatic cancer (Liszka et al., 2011). These findings are consistent with the idea that PanIN lesions are precursors of pancreatic adenocarcinoma, thereby rising important questions about the mechanisms of progression from PanIN to cancer. Since PanIN lesions display markers of cellular senescence, their progression to cancer must bypass this tumor suppressor mechanism. To understand the changes associated with the transition of premalignant to malignant lesions, we developed in vitro models of different stages of pancreatic cancer progression. For early‐stage disease, we established pancreatic epithelial cell lines from *Pdx1‐Cre;LSL‐Kras^G12D^*mice harboring low‐grade PanIN1 lesions and regions of acinar‐ductal metaplasia from which PanINs originate (Figure 1a). To model the transition from PanIN to PDAC, we used the PanIN‐derived AH375 cell line, which evolved into invasive PDAC upon passaging in vitro. We also established a PDAC cell line from *Pdx1‐Cre;LSL‐Kras^G12D^*; p53^flox/+^ mice bearing aggressive tumors (NB508). The ADM/PanIN‐derived cell lines (1,497, 1,498, 1,499) had lower proliferation rates relative to the PDAC cell lines (NB508 and AH375) (Figure 1b). Only PDAC cells formed colonies in soft agar (Figure 1c,d) and tumors in vivo (Figure 1e–g). On the other hand, PanIN cells exhibited markers of cellular senescence such as senescence‐associated β‐galactosidase (Figure 1h and Supporting Information Figure S1a), DNA damage foci (Figure 1i), and increased staining for the heterochromatin marker HP1‐γ (Rielland et al., 2014; Figure 1j). Since these cells still grow in culture, they likely represent a mixture of senescent and pre‐senescence cells (Itahana et al., 2003). We also compared expression of several pro‐inflammatory genes between PanIN cells and PDAC cells. We found that the expression of Angptl2, Il‐23a, Ccl5, Il‐15, and Il‐1α was increased in PanIN cells, while the expression of Ccl7 was decreased (Supporting Information Figure S1b,c). These genes are often upregulated in senescent cells as part of the senescence‐associated secretory phenotype (SASP; Coppe et al., 2008). Further evidence for a senescence gene expression pattern of PanIN cells was obtained using novel senescent biomarkers common to multiple cell types (Hernandez‐Segura et al., 2017). Again, PanIN cells show gene expression changes corresponding to the senescent phenotype (Supporting Information Figure S1d). Overall, these gene expression changes and senescence biomarkers that are common to other senescent cells support the idea that PanIN cells are under oncogenic stress.

**Figure 2:**
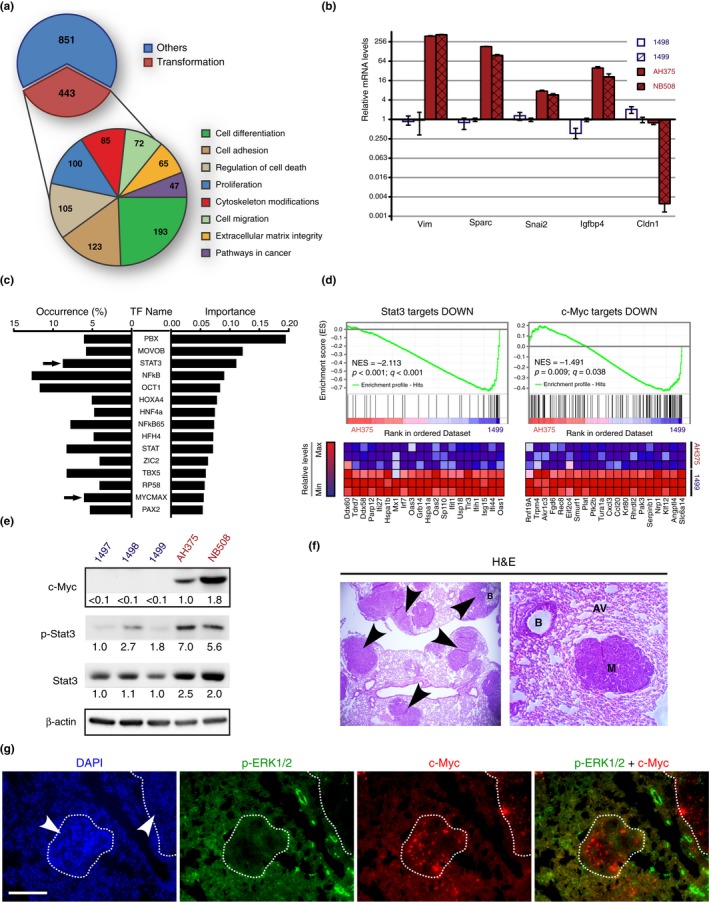
Stemness gene expression pattern in pancreatic ductal adenocarcinoma cells. (a) Transcriptome analysis comparing 1,499 and AH375 cells (GEO accession number: GSE57566). Transcripts with a fold change higher or equal to 2 and a *p* < 0.05 according to a two‐sample Student's *t* test were analyzed with the Babelomics 4.3 platform. The number of transcripts in each category (nonmutually exclusive) is indicated. (b) Validation by qPCR of the microarray data in the indicated cell lines and for the indicated genes, which are involved in epithelial‐mesenchymal transition (EMT). Mean of triplicates ± *SD*. (c) DIRE prediction of upregulated transcription factors (TF) in AH375 cells. The percentage of target genes found in the submitted list of transcripts is shown for each potential TF (occurrence). The importance indicates the product of a TF occurrence with its weight in the database. (d) GSEA found gene expression signatures suggesting upregulation of Stat3 and c‐Myc in AH375 cells. (e) Immunoblots with anti‐phospho‐Stat3 (Y705), anti‐Stat3, and anti‐c‐Myc antibodies on extracts from the indicated cell lines. (f) Histology of lung tumors formed after tail vein injection of 1 × 10^6^ AH375 cells into SCID mice. B, bronchiole; AV, alveolus; M and black arrows, tumors. H&E, hematoxylin and eosin (g) Indirect immunofluorescence staining with anti‐Myc and anti‐phospho‐ERK antibody of mouse lung tissues containing tumors as in (f). White arrows, metastasis; DAPI, nuclear counter stain; scale bar = 100 μm

To better characterize gene expression changes distinguishing PanIN from PDAC, we compared the gene expression profile of the 1,499 ADM/PanIN1‐derived cell line, with that of AH375 PDAC cells. Using a FatiGO single enrichment analysis of microarray data from the Babelomics 4.3 platform, we found marked differences in the general biological function categories associated with cellular transformation, with most pronounced changes in genes regulating cell differentiation (Figure 2a). In particular, compared to 1,499 cells, the AH375 cell line had enrichment of stem cell genes and lower levels of genes associated with cell differentiation (Supporting Information Figure S2a,b). AH375 cells also exhibited features of epithelial‐mesenchymal transition (EMT), which is often associated with acquisition of stem cell traits (Figure 2b and Supporting Information Figure S2a,b) (Polyak & Weinberg, 2009). Overall, our analysis suggests that malignant progression of PanINs correlates with a reprogrammed gene expression profile associated with dedifferentiation, stem cell features, and EMT.

To evaluate the mediators of this transcriptional reprogramming, we analyzed the promoters of the significantly regulated genes for known transcription factor binding sites using the DiRE platform. Strikingly, several transcription factors with reported roles in differentiation or stemness were found among the most significant candidates (Figure 2c) including STAT3 (Yang et al., 2010), c‐MYC/MAX (Dang, 2012; Wong et al., 2008), and NF‐κB (Sun et al., 2013). Consistently, gene expression signatures suggested downregulation of Stat3 and c‐Myc functions in 1,499 cells (Figure 2d). The data also implicated additional key signaling pathways associated with stem cells and regulation of c‐Myc and Stat3, including the LIF/STAT3 pathway (Supporting Information Figure S2a) (Yang et al., 2010), and WNT signaling (Supporting Information Figure S2c) (Clevers & Nusse, 2012). Intriguingly, while 1,499 cells expressed many genes regulated by NF‐κB, AH375 cells poorly express genes downregulated by the same transcription factor (Supporting Information Figure S2c) suggesting a reprogramming of the NF‐κB pathway and a role for NF‐κB‐mediated gene repression in pancreatic cancer. In line with this result, it was reported that NF‐κB‐mediated gene repression was important to protect cancer from cytotoxic stimuli (Campbell, Rocha, & Perkins, 2004). Next, we confirmed that c‐Myc and Stat3 protein levels were increased in the PDAC cell lines compared to the PanIN lines (Figure 2e). We confirmed the robust expression of c‐Myc and low relative levels of pErk in vivo in lung tumors derived from tail vein‐injected AH375 cells (Figure 2f,g). Of note, low p‐ERK levels characterize embryonic stem cells and facilitate the reprogramming of somatic cells into pluripotent stem cells (Silva et al., 2008). These findings further link PDAC progression with the acquisition of more primitive transcriptional states and stemness phenotypes.

### 2.2 Mouse and human cells that circumvent OIS express stemness genes

**Figure 3:**
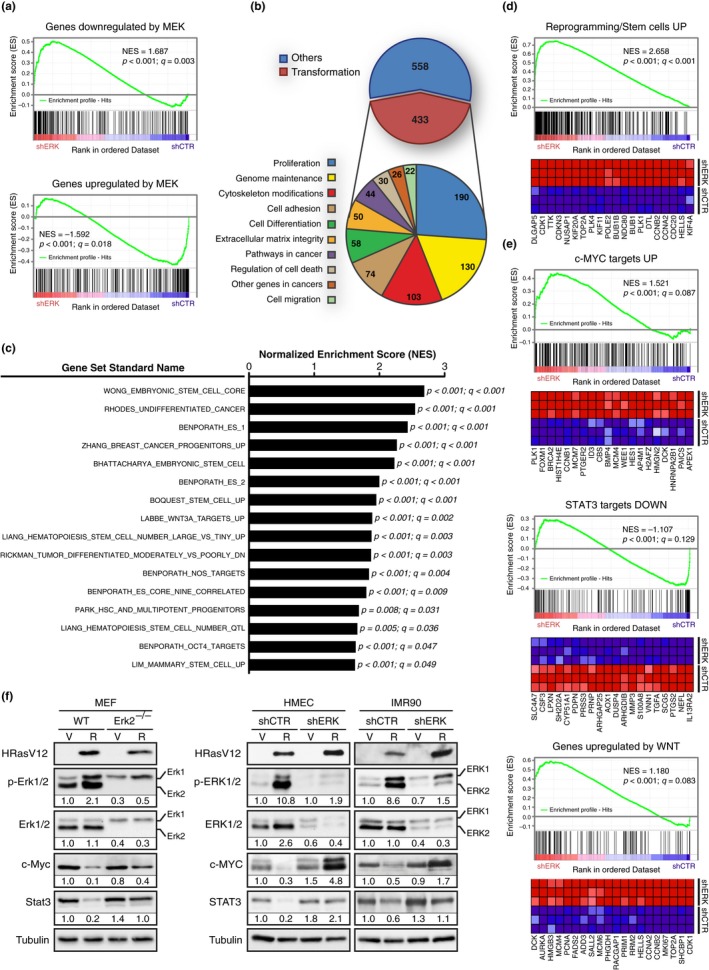
Ras‐transformed mouse embryonic fibroblasts (MEFs) and human primary cells show a reprogrammed gene expression profile associated with dedifferentiation. (a) RNA from IMR90 cells stably expressing hTERT, HRasG12V, and a shRNA against ERK2 (shERK) or a non‐targeting shRNA (shCTR) was collected for microarray gene expression analysis (GEO accession number: GSE33613). GSEA revealed gene expression signatures of genes known as downregulated by active MEK in cells expressing a shERK2 (MEK UP.V1 DN; M2724) and genes known as upregulated by active MEK in cells expressing a shCTR (MEK UP.V1 UP; M2725). (b) A total of 991 transcripts with a fold change higher or equal to 2 and a *p* < 0.05 following a two‐sample Student's *t* test were analyzed with the Babelomics 4.3 platform. The terms obtained which may explain the transformed phenotype of the IMR90 hTERT/HRasG12V/shERK2‐expressing cells and their associated transcripts are grouped in the indicated general categories. The number of transcripts in each category (nonmutually exclusive) is indicated. (c) Several stem cell gene expression signatures showing dedifferentiation in IMR90 hTERT/HRasG12V/shERK2‐expressing cells were revealed by GSEA. (d) GSEA for the most significant signature in (c) (WONG EMBRYONIC STEM CELL CORE; M7079). (e) Gene expression signatures suggesting upregulation of STAT3, c‐MYC, and the WNT pathway in IMR90 hTERT/HRasG12V/shERK2‐expressing cells. (f) Immunoblots for the indicated proteins in extracts from wild‐type (WT) or Erk2‐null MEF, and IMR90 or human mammary epithelial cell (HMEC) cells expressing hTERT and the indicated vectors: shCTR, nontargeting shRNA; shERK, shRNA targeting ERK2; V, empty vector; R, vector expressing HRasG12V

To investigate the bypass from OIS to transformation more broadly, we utilized normal human lung fibroblast IMR90, primary human mammary epithelial cell (HMEC), and primary mouse embryonic fibroblast (MEF) models of OIS. In each model, shRNA‐mediated inhibition of aberrant ERK signaling promotes bypass of senescence allowing tumor formation in vivo, consistent with prior studies (Supporting Information Figure S3) (Deschenes‐Simard et al., 2013). We compared the transcriptome of senescent human fibroblasts expressing oncogenic Ras with that of the same cells that circumvented senescence due to knockdown of ERK2 (Deschenes‐Simard, Roy, & Ferbeyre, 2016). In addition to the expected decrease in signatures for the RAS‐ERK MAP kinase pathway, (Figure 3a,b), cells that bypassed senescence expressed high levels of stem cell genes (Figure 3c,d), including targets of MYC, STAT3, and the WNT pathway (Figure 3e). In both IMR90s and HMECs, the levels of MYC and STAT3 dropped sharply with senescence and were fully restored upon senescence bypass due to ERK2 inactivation (Figure 3f). We suggest that bypass from senescence provides a context where these transcription factors can drive the expression of their target genes over that found in the control cells. Collectively, these results relate bypass from RAS‐induced senescence to acquisition of a “stemness” gene expression program. Of note, the stemness signature as defined using GSEA and other bioinformatic programs is not specific for stem cells since they were derived from mixed populations such as the cell lines used in the current report. It is likely that the progeny of stem‐like cells expresses genes controlled by key stemness factors such as Myc and Stat3 that remained activated in the stem cell progeny. Hence, these gene expression correlations only suggest but do not proof stemness.

### 2.3 Bypass from senescence is associated with the emergence of cells with stemness properties

**Figure 4:**
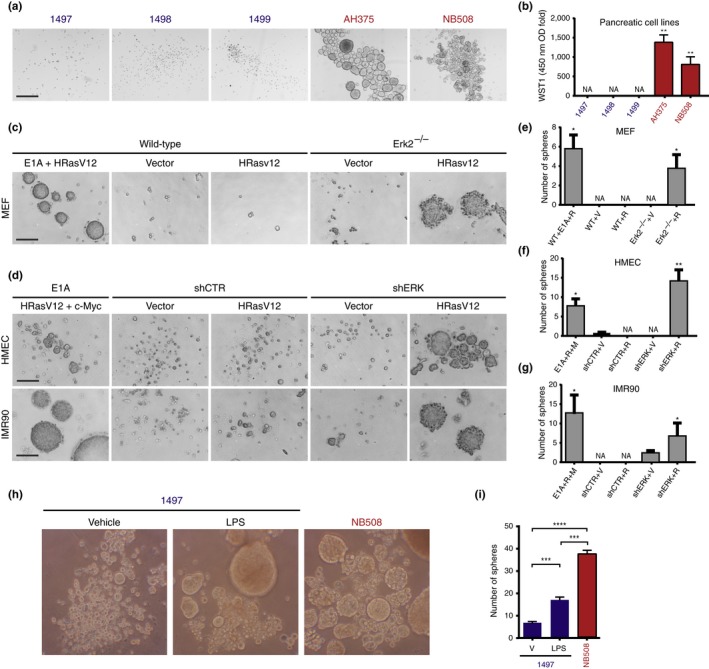
A subpopulation of cells that bypass senescence have a behavior of cancer stem cells. (a) Cell lines from pancreatic ductal adenocarcinoma form free‐floating tumor spheres, but not cell lines from ADM/PanIN1 lesions. Scale bar = 400 μm. (b) Quantification of cell proliferation in (a) with the WST‐1 cell proliferation assay. The fold of absorbance at 450 nm over a period of 14 days is shown. Average of triplicates ± *SD*, **p < 0.01. (c) Tumor sphere formation assay with wild‐type or Erk2‐null mouse embryonic fibroblast (MEF) expressing the indicated vectors. Scale bar = 200 μm. (d) Tumor sphere formation assay with human mammary epithelial cell (HMEC) and IMR90 cells expressing hTERT and the indicated vectors. shCTR: control nontargeting shRNA, shERK: shRNA against ERK2. Scale bar = 200 μm. (e–g) Number of spheres from an initial plating of 1,000 cells; (e) MEF, (f) HMEC, (g) IMR90. Average of triplicates ± *SD*, *p < 0.05, **p < 0.01 versus WT + V or NTC + V. (h) LPS stimulates the conversion of PanIN cells into tumor sphere‐forming cells. Representative pictures of spheres formed by LPS‐ or vehicle‐pretreated 1,497 cells and of NB508 cells grown in suspension. Note that LPS pretreatment was performed on adherent cells, whereas no LPS was added in culture medium when LPS‐pretreated cells were seeded in suspension (i) Quantification of sphere number. Average of six replicates ± *SD*, ***p < 0.001, ****p < 0.0001, V = vehicle

To determine whether our findings at the gene expression level are transposable to a phenotype of CSCs, we tested the capacity of the mouse cell lines derived from the different pancreatic lesions to form free‐floating tumor spheres. It was previously shown that both normal (Reynolds & Weiss, 1996) and cancer stem cells (Hirsch, Iliopoulos, Tsichlis, & Struhl, 2009; Singh et al., 2003) form spheres in 3D culture conditions. While PanIN‐derived cell lines failed to form spheroids, PDAC cells did so readily, thereby revealing the presence of a subpopulation of tumor‐initiating cells in these cell lines (Figure 4a,b). In addition, fibroblasts and HMEC that bypassed Ras‐induced senescence due to expression of a shRNA against ERK2 were also capable of forming tumor spheres (Figure 4c–g). Taken together, our results show an intriguing link between bypassing senescence and the acquisition of stem cell‐like properties.

The malignant pancreatic cancer cell line AH375 spontaneously developed from cells obtained from PanIN lesions. It is commonly assumed that mutations in tumor suppressor pathways contribute to circumvent senescence and to the conversion of benign PanIN lesions into PDAC. However, activation of pattern recognition receptors (PRRs) can accelerate tumorigenesis via immune suppression and activation of protumorigenic signaling pathways that include NF‐κB, Notch, and STAT3 (Seifert et al., 2016; Zambirinis et al., 2013). The Notch and Stat3 pathways are clearly activated in PDAC AH375 cells that spontaneously arose from PanIN cell cultures and the NF‐κB pathway seems to be reprogrammed toward repression in AH375 cells (Figure 2 and Supporting Information Figure S2). To investigate whether activation of PRRs can stimulate the conversion of PanIN cells into PDAC in culture, we treated 1,497 PanIN cells with LPS and tested their ability to form tumor spheres. Consistent with a protumorigenic role of PRR activation, LPS promoted the formation of tumor spheres by 1,497 cells (Figure 4h,i).

### 2.4 Pancreatic cancer cells show increased mitochondrial machinery

**Figure 5:**
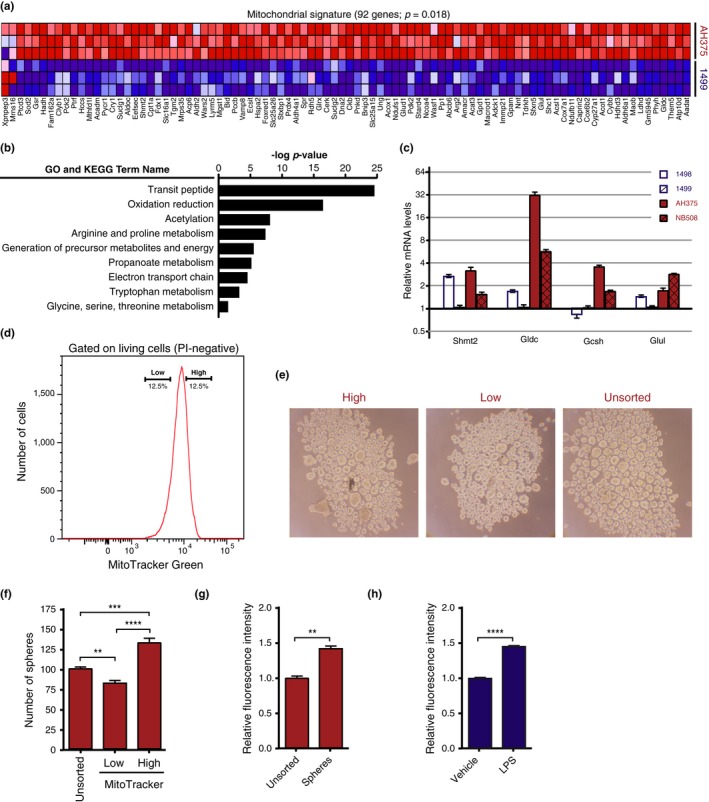
Increased mitochondrial gene expression in pancreatic cancer cells. (a) An analysis of the microarray data as in Figure 2 (AH375 vs. 1,499 cells) with DAVID 6.7 revealed a mitochondrial gene expression signature (92 genes). (b) The mitochondrial gene expression signature found by DAVID 6.7 regrouped the indicated GO and KEGG terms. (c) QPCR validation of the indicated genes in AH375 and NB508 pancreatic ductal adenocarcinoma cells in comparison with 1,498 and 1,499 PanIN cells. Error bars represent mean ± *SD*. (d) Sorting AH375 cells based on MitoTracker Green staining. (e) Representative tumor spheres of unsorted AH375 or cells sorted according to MitoTracker Green staining as in (d). (f) Quantification of AH375 cells tumor sphere formation of unsorted or sorted subpopulations with MitoTracker Green low versus high as in (d). Average of triplicates ± *SD*, **p < 0.01, ***p < 0.001, ****p < 0.0001 using ANOVA. (g) Flow cytometry quantification of MitoTracker Green staining of AH375 cells growing in 2D culture or as tumor spheres (3D culture). Average of triplicates ± *SD*, **p < 0.01 using Student's *t* test. (h) Flow cytometry quantification of relative MitoTracker Green staining median intensity in LPS‐ or vehicle‐pretreated 1,497 cells. Average of triplicates ± *SD*, ****p < 0.0001 using Student's *t* test

Another striking feature of the transcriptome of PDAC cells in comparison with PanIN cells is an increase in the expression of mitochondrial genes (Figure 5a,b). High mitochondrial mass correlates with cancer stem cell properties (Farnie, Sotgia, & Lisanti, 2015), and in pancreatic tumors, the stem cell population has an increase in respiration and dependency on electron transport and oxidative phosphorylation (Sancho et al., 2015; Viale et al., 2014). Also, RAS‐dependent transformation requires mitochondrial STAT3 (Gough et al., 2009) a protein that is compromised in PanIN cells and OIS cells. We found that many mitochondrial enzymes involved in amino acid metabolism were upregulated in PDAC cells in comparison with PanIN cells (Figure 5a,b). These include several enzymes in one‐carbon metabolism pathways such as serine hydroxymethyltransferase (Shmt2) (Lee et al., 2014) and glycine decarboxylase (GLDC) (Hiraga & Kikuchi, 1980). Notably, GLDC is important for the growth of tumor‐initiating cells in lung cancer (Zhang et al., 2012). We confirmed by qPCR analysis that Gldc and Gcsh were elevated in PDAC cells compared to PanIN cells (Figure 5c). These results are in keeping with the reported role for mitochondrial biogenesis in the growth of PDAC (Sancho et al., 2015; Viale et al., 2014).

The cells capable of initiating tumors within most cell lines are often a fraction of the total cell population. The stem cell hypothesis of cancer proposes that tumor‐initiating cells are for the most part quiescent but produce a progeny of proliferating cells where many cells cannot form tumors due to cell differentiation (epigenetic changes) or accumulation of maladaptive genetic changes (Kreso & Dick, 2014). To investigate the frequency of tumor‐initiating cells in AH375 cells, we injected serial limiting dilutions of the cells (10,000, 1,000, 100 cells) subcutaneously in SCID mice and scored the number of visible tumors for each injection. The data were analyzed with extreme limiting dilution analysis (ELDA) (http://bioinf.wehi.edu.au/software/elda/). We found that 1/2,473 cells were capable of initiating tumors (Supporting Information Figure S4a). We also identified quiescent stem‐like cells in AH375 cells growing as tumor spheres using a label retention assay with CFSE. AH375 cells were stained and plated for tumor spheres. After 3 days in culture, each sphere displayed a single cell labeled with CFSE (Supporting Information Figure S4b) suggesting that putative cancer stem cells divided only once asymmetrically and then remained quiescent.

To gain more insight into the heterogeneity of AH375 PDAC cells, we sorted the cells according to mitochondrial mass using MitoTracker Green (Figure 5d). We found that cells with higher mitochondrial content formed more tumor spheres than cells with lower mitochondrial content (Figure 5e,f). Also, the mitochondrial content of the cells that formed spheres was higher than that of cells growing in adherent conditions (Figure 5g). In addition, treatment of PanIN 1,497 cells with LPS stimulated their conversion into tumor sphere‐forming cells (Figure 4h,i) and increased their mitochondrial content (Figure 5h). Taken together, our results show an association between mitochondrial genes and mitochondrial mass with the transition from PanIN to PDAC, a process that is stimulated by LPS.

### 2.5 Metformin targets reprogrammed pancreatic cancer cells

**Figure 6:**
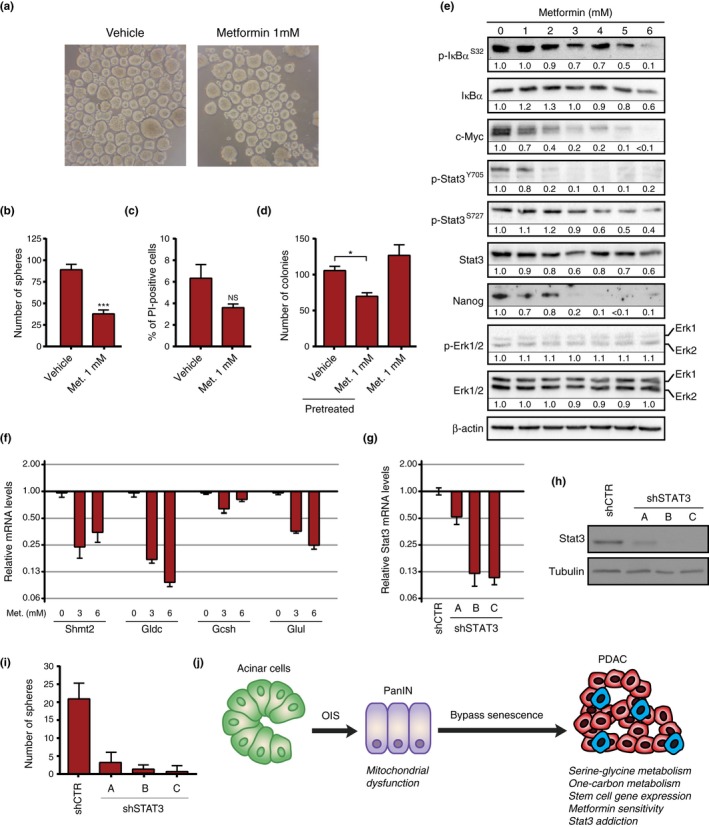
Pretreatment with metformin reduces the ability of AH375 pancreatic ductal adenocarcinoma (PDAC) cells to form tumor spheres. (a) AH375 cells pretreated with metformin or vehicle. (b) Quantitation of tumor spheres as in (a). Average of replicates ± *SD*, ***p < 0.001, *n* = 3. (c) Quantitation of AH375 cell viability using propidium iodide (PI) after treatment with metformin or vehicle. NS = not significant. (d, e) Immunoblots for the indicated proteins in extracts from AH375 cells treated with the indicated concentrations of metformin for six days. (f) QPCR for mitochondrial genes in AH375 cells treated with the indicated doses of metformin for 5 days. Error bars indicate mean ± *SD*. (g) QPCR for Stat3 expression in NB508 expressing a nontargeting control shRNA (shNTC) or different shRNAs against Stat3. (h) Immunoblots for Stat3 and tubulin as in (g). (i) Number of tumor spheres in cells as in (g) after 5 days. Spheres were counted by microscopic examination. Average of quadruplicate ± *SD*. (j) Bypassing senescence and mitochondrial reprogramming in carcinogenesis. Premalignant lesions are first halted by oncogene‐induced senescence, but some cells can bypass the process. This involves an enrichment for stemness genes, mitochondrial genes, and a subpopulation of metformin‐sensitive tumor‐initiating cells (blue cells in the figure)

The increased mitochondrial content of pancreatic cancer cells suggested that they might be sensitized to drugs that target mitochondria such as metformin (Owen, Doran, & Halestrap, 2000). The proportion of cancer stem‐like cells in the population of AH375 cells growing in 2D is likely low. However, if these cells require an increase in mitochondrial function while they are growing in 2D, a pretreatment with metformin before plating them for the tumor sphere 3D assay should reduce the number of tumor spheres. We found that a pretreatment with 1 mM metformin for three days was sufficient to reduce by almost 50% the number of tumor spheres formed by AH375 cells (Figure 6a,b) while having a minimal effect on cell viability (Figure 6c). Together, the results suggest that metformin could be a valuable drug to target mitochondria in cancer stem cells. Metformin also inhibited AH375 cells from forming colonies in soft agar (Supporting Information Figure S5a,b) and from growing as tumor spheres (Supporting Information Figure S5a,c). Metformin reduced sphere formation of NB508 PDAC cells but at higher concentration (Supporting Information Figure S5d). Similar results were seen in transformed MEF and IMR90 cells (Supporting Information Figure S5e,f) and in the human pancreatic cancer cell line HPAF‐II (Supporting Information Figure S5g).

To get insights into the mechanisms by which metformin inhibits stemness, we measured the levels of several transcription factors required for the stem cell‐like phenotype. We found that metformin reduced the levels of Nanog, c‐Myc, and phosphorylated Stat3 (Figure 6d). Metformin also induced a gene expression pattern characteristic of a more epithelial state (Figure 6e). An inhibition of the NF‐κB pathway was also observed at higher concentrations, as seen by a decrease of IκBa phosphorylation (Figure 6d). Interestingly, treatment of AH375 PDAC cells with 3‐ or 6‐mM metformin reduced the expression of Shmt2, Gldc, Gcsh, Glu1, and EMT genes (Figure 6e and (Supporting Information Figure S6). As an example that the comparisons of transcriptomes of premalignant cells with tumor cells and the use of metformin can identify stemness factors in cancer cells, we knocked down Stat3 in NB508 PDAC cells. Depletion of Stat3 drastically inhibited tumor sphere formation in these cells (Figure 6g–i).

## 3 DISCUSSION

We show here that bypass of OIS was associated with the acquisition of a gene expression program characteristic of stem cells, EMT, and mitochondrial genes. This gene expression transition was observed after comparing premalignant pancreatic intraepithelial neoplasias (PanINs) with pancreatic ductal adenocarcinoma cells that spontaneously arose from PanIN cells. Also, these results were reproduced in other models of OIS in primary human and mouse cells. We found that PanIN cells that progress into PDAC show enrichment for genes regulated by known drivers of CSCs such as NF‐κB (Sun et al., 2013), STAT3 (Yang et al., 2010), and MYC (Sancho et al., 2015). These cells also acquire properties of tumor‐initiating cells including the ability to form tumor spheres in cell culture and ectopic tumors in vivo by extravasation. The protumorigenic pathways activated in cells that bypass senescence can be also stimulated by activation of PRRs that play an important role in the origin of pancreatic cancer (Zambirinis et al., 2013). Finally, the subpopulation of tumor‐initiating cells in PDAC cells show increased mitochondrial content and are sensitized to metformin. Taken together, these observations suggest that OIS acts as an initial barrier for the proliferation of cells with oncogenic mutations but seems to prime cells to activate a stemness gene expression program that can contribute to progression into malignant tumors.

### 3.1 The transition from PanIN

Two models can explain the protumorigenic actions of cellular senescence. First, senescent cells secrete a variety of inflammatory factors that have been previously linked to tumorigenesis in a noncell autonomous manner and stimulate the progression of cells that have not fully entered senescence (Coppe, Desprez, Krtolica, & Campisi, 2010). Consistent with this idea, the histone deacetylase‐associated protein SIN3B is required for Kras‐induced senescence, secretion of IL‐1α, and progression to PDAC in the pancreas. Also, SASP factors induce stemness and proregenerative responses in the skin of mice (Ritschka et al., 2017). However, other studies show a tumor suppressor role for the SASP. For example, inactivation of RelA or the TGFβ pathway in mouse models of Kras‐driven pancreatic cancer accelerated pancreatic cancer formation by inhibiting the SASP (Acosta et al., 2013; Lesina et al., 2016). In the liver, the SASP also plays a role in stimulation of antitumor immune responses (Iannello, Thompson, Ardolino, Lowe, & Raulet, 2013; Lujambio et al., 2013).

A second model proposes that some senescent cells can evolve into tumor cells in a cell autonomous manner. In this case, intrinsic properties of the cells are important for tumor progression. Peeper and colleagues showed that inhibition of IL‐6 expression could reverse human fibroblasts from RAF‐induced senescence (Kuilman et al., 2008). Abbadie and colleagues have described an interesting mechanism in which rare senescent epithelial cells can give rise to a progeny of tumorigenic cells via amitotic cell division similar to budding (Gosselin et al., 2009). This process happens with a very low frequency and is stimulated by single‐stranded DNA breaks (Gosselin et al., 2009). Recently, Schmitt and colleagues reported that senescent cells induced by chemotherapy in hematopoietic tumor cells express stemness genes and if they manage to overcome the cell cycle arrest they progress into very aggressive tumors (Milanovic et al., 2018). Further work will be required to find whether malignant tumors arise from fully senescent cells that escape from the arrest or from cell expressing the driving oncogene that avoided senescence due to factors in the microenvironment. The two models presented above (noncell autonomous vs. cell autonomous) are not mutually exclusive since it is plausible that specific inflammatory factors can act in synergy with cell intrinsic mechanisms that promote escape from senescence.

An important question still unresolved is the identity of the proinflammatory factors that can help to circumvent senescence. In a mouse model of Kras‐driven pancreatic cancer, pancreatitis was required for PanIN lesions to progress to PDAC in association with inhibition of senescence (Guerra et al., 2011). This study suggests that the factors secreted by PanIN cells are not sufficient to promote malignant progression and that other factors controlled by the microenvironment are important for tumorigenesis. The microbiota plays an important role in the origin of pancreatic cancer (Farrell et al., 2012; Pushalkar et al., 2018). Consistent with these results, we show here that LPS, a bacterial product, can stimulate the emergence of cells with stem cell properties from PanIN cells. Future investigation of the effect of LPS on PanIN cells can help to identify critical factors that affect their senescent phenotype. Of note, while senescence and LPS both involve increased NF‐κB signaling, they differ on the status of the STAT3 pathway. During senescence, STAT3 is downregulated via proteasome‐dependent protein degradation (Deschenes‐Simard et al., 2013) and its phosphorylation is reduced in PanIN cells (Figure 2e). LPS can potentially change the SASP by activating STAT3 signaling (Lin et al., 2016), which can synergize with NF‐κB, thereby changing gene expression patterns and promoting tumorigenesis (Grivennikov & Karin, 2010). In addition, STAT3 can promote mitochondrial functions required for cancer cell stemness and metabolism (Genini et al., 2017). IL‐6 activates STAT3 and promotes the development of PDAC from PanIN (Lesina et al., 2011). Although this cytokine is required to maintain OIS in fibroblasts, it could have a different role in the pancreas linking LPS to STAT3 signaling and senescence bypass.

The biology of senescent cell populations can provide clues for the long‐sought mechanism to explain why most malignant tumors occur in old individuals. Pre senescent and senescent cells seem primed to reactivate stem cell programs and mitochondrial genes that confer specific survival advantages (Figure 6j). Fortunately, cells that emerge from lesions containing senescent cells are sensitive to the antimitochondrial drug metformin. The sensitivity to metformin in pancreatic cancer stem cells was explained by an intrinsic inability of these cells to turn on glycolysis upon inhibition of oxidative phosphorylation (Sancho et al., 2015; Viale et al., 2014). In contrast, normal cells and the bulk of tumor cells (non‐CSCs) survive inhibition of oxidative phosphorylation due to their ability to increase glycolysis. Of note, senescent cells are highly glycolytic (Moiseeva, Bourdeau, Roux, Deschenes‐Simard, & Ferbeyre, 2009) suggesting that the defects in this pathway observed in cancer stem cells are acquired during tumor progression. Metformin treatment of mice expressing oncogenic Kras in the pancreas delayed tumorigenesis and decreased the percentage of both early and late PanIN lesions (Chen et al., 2017). Also, metformin has been shown to target selectively breast and pancreatic cancer cells with stem cell properties (Hirsch et al., 2009; Sancho et al., 2015). These results are consistent with a requirement for mitochondria in cells capable of initiating tumor growth. Notably, cancer chemotherapy can induce tumor cell senescence with the potential risk that some cells escape from the process with stem cell properties (Milanovic et al., 2018). We suggest that metformin could be considered as a general adjuvant to prevent tumor regrowth from cells that acquired stemness induced by the treatment.

## 4 MATERIALS AND METHODS

### 4.1 Mouse experiments

All mouse experiments were approved by the University of Montreal animal ethics committee (CDEA) or the subcommittee on research animal care of the Massachusetts General Hospital. For subcutaneous xenografts, 5 × 10^5^ or 1 × 10^6 ^of the specified cells were suspended in 50 µl PBS mixed with 50 µl Matrigel (#356234; BD Biosciences) and then injected subcutaneously into the lower flank of SCID mice (C3SnSmn.CB17‐Prkdcscid/J; Jackson Labs) or BALB/c nude mice (CAnN.Cg‐*Foxn1* ^nu^/Crl; Charles River). Mice were euthanized before the end point of the experiment when tumor volume reached 2 cm^3^. Tumors were weighed, their volumes were calculated according to the formula (length × width^2^)/2, and were fixed in formalin for paraffin embedding and then staining with hematoxylin and eosin (H9627; Sigma).

For orthotopic xenografts into the pancreas, SCID mice were subjected to general anesthesia with intraperitoneal injection of avertin 0.5 mg/g. A left lateral laparotomy was performed in order to visualize the spleen and then mobilize the distal pancreas. Approximately 100 µl of cell suspension (50 µl PBS and 50 µl Matrigel) containing 5 × 10^5^ AH375 cells were injected into the pancreas. The abdominal incision was closed using silk suture 3/0 (K943H; Ethicon) for the peritoneum and the skin. The mice were inspected daily for a week for any health complication and were examined for tumor formation by palpation. Mice were euthanized after a maximum of 21 days, and pancreas was fixed in formalin for paraffin embedding and staining.

For tail vein assay of cancer metastasis, SCID mice were injected with a suspension of 1.25 × 10^6^ cells/ml was prepared in divalent cation‐free PBS. The mice were placed in a restrainer and the tails were immerged in warm water (50–60°C) for 2 min to dilate the veins. Then, 200 µl of the cell suspension was injected into the lateral tail vein using a 3/10 cc syringe with a 29G½’’ needle. Mice were euthanized 3 weeks after injection and lungs were collected and fixed in formalin for paraffin embedding and staining.

### 4.2 Cell culture and viral‐mediated gene transfer

Human cancer cell lines were obtained from the American Type Culture Collection (ATCC) or from the Massachusetts General Hospital Center for Molecular Therapeutics (CMT). IMR90 was obtained from ATCC and transduced with pBABE hTERT for this study. HMEC is from Lonza. MEFs were a kind gift of Dr. Sylvain Meloche (IRIC, University of Montréal, Qc, Canada). 1,497, 1,498, and 1,499 pancreatic ductal cell lines were isolated from 9‐week‐old *Pdx1‐Cre;LSL‐Kras^G12D^* mice as reported previously (Corcoran et al., 2011). The transformed AH375 pancreatic ductal cell line was established from a 52‐week‐old *Pdx1‐Cre;LSL‐Kras^G12D^* mouse. The procedure used for isolation and growth of 1,497, 1,498, 1,499 and AH375 cells is detailed in the Supporting Information Data S1. For LPS‐mediated conversion of PanIN cells into PDAC cells, PanIN 1,497 cells grown as adherent monolayers were treated for four weeks with 4 μg/ml lipopolysaccharide (LPS) from *E. coli* (Sigma‐Aldrich #L2880) in water or with vehicle (water). Culture medium and treatments were renewed every 2–3 days.

Retroviral and lentiviral‐mediated gene transfers were done as described (Deschenes‐Simard et al., 2013). For senescence bypass experiments, retrovirus expressing RasV12 and shERK2 were simultaneously used to infect target cell populations. Cell proliferation assays, SA‐β‐Gal, soft agar assays, sphere‐forming assays, immunoblotting procedure, immunofluorescence staining protocol, reagents, and plasmids are described in the Data S1.

### 4.3 Flow cytometry and MitoTracker Green staining

AH375 cells grown adherent or in suspension (tumor spheres) were trypsinized and stained with MitoTracker Green (Thermo Fisher) at 1/7,500 for 10 min at 37°C in PBS‐EDTA 5 mM with 2% fetal calf serum. Cells were centrifuged and washed twice with 10 volumes of PBS‐EDTA 5 mM with 2% fetal calf serum. Flow cytometry analysis was performed on a Canto II flow cytometer. MitoTracker Green fluorescence was detected in the GFP channel, gated on PI (propidium iodide) negative single cells. Cell sorting was performed at IRIC cytometry platform on a BD FACSAria high‐speed cell sorter.

### 4.4 Statistical analysis

Student's *t* tests or ANOVA were used for comparisons between groups based on an assumption of normal distribution. Significant differences were considered at *p < 0.05, **p < 0.01, ***p < 0.001. Results are represented as means ± standard deviation (*SD*).

## CONFLICT OF INTEREST

None declared.
