# Supplementary figures and images for: Circumventing senescence is associated with stem cell properties and metformin sensitivity
Source: Aging Cell. 2019 Jan 6;18(2):e12889. doi: 10.1111/acel.12889 (PMC6413657; doi:10.1111/acel.12889)

(a)

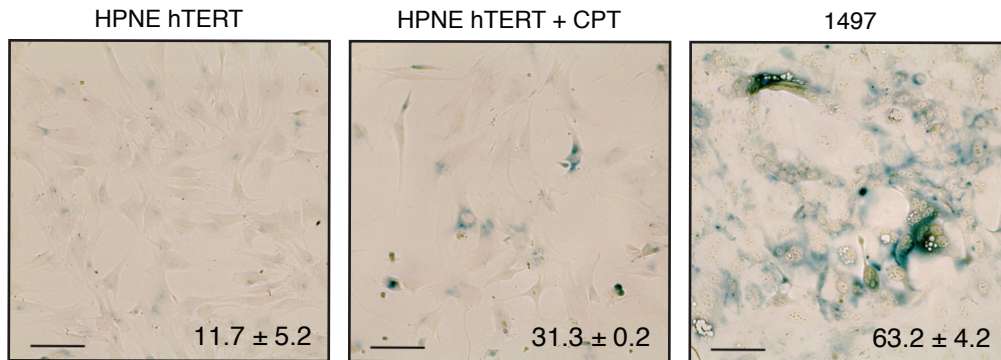

(b)

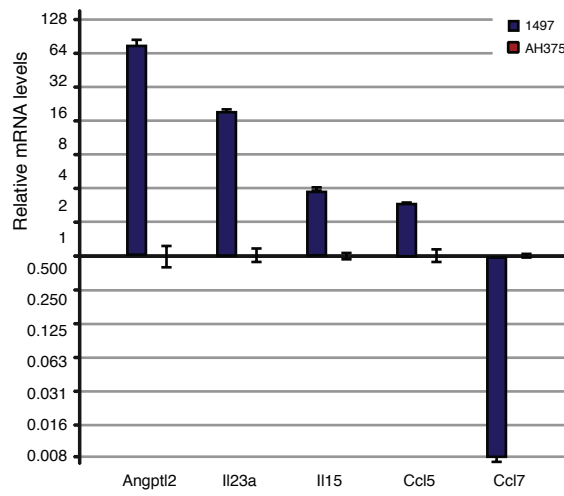

(c)

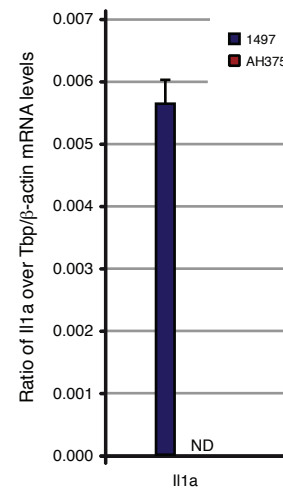

(d)

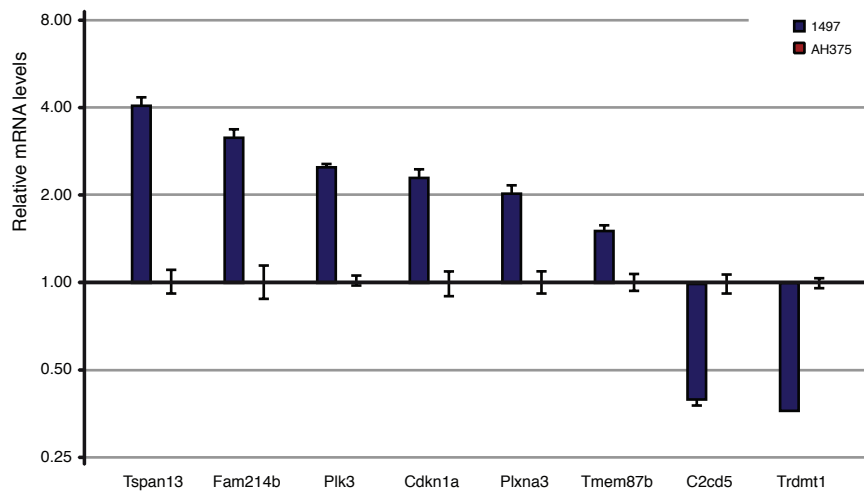

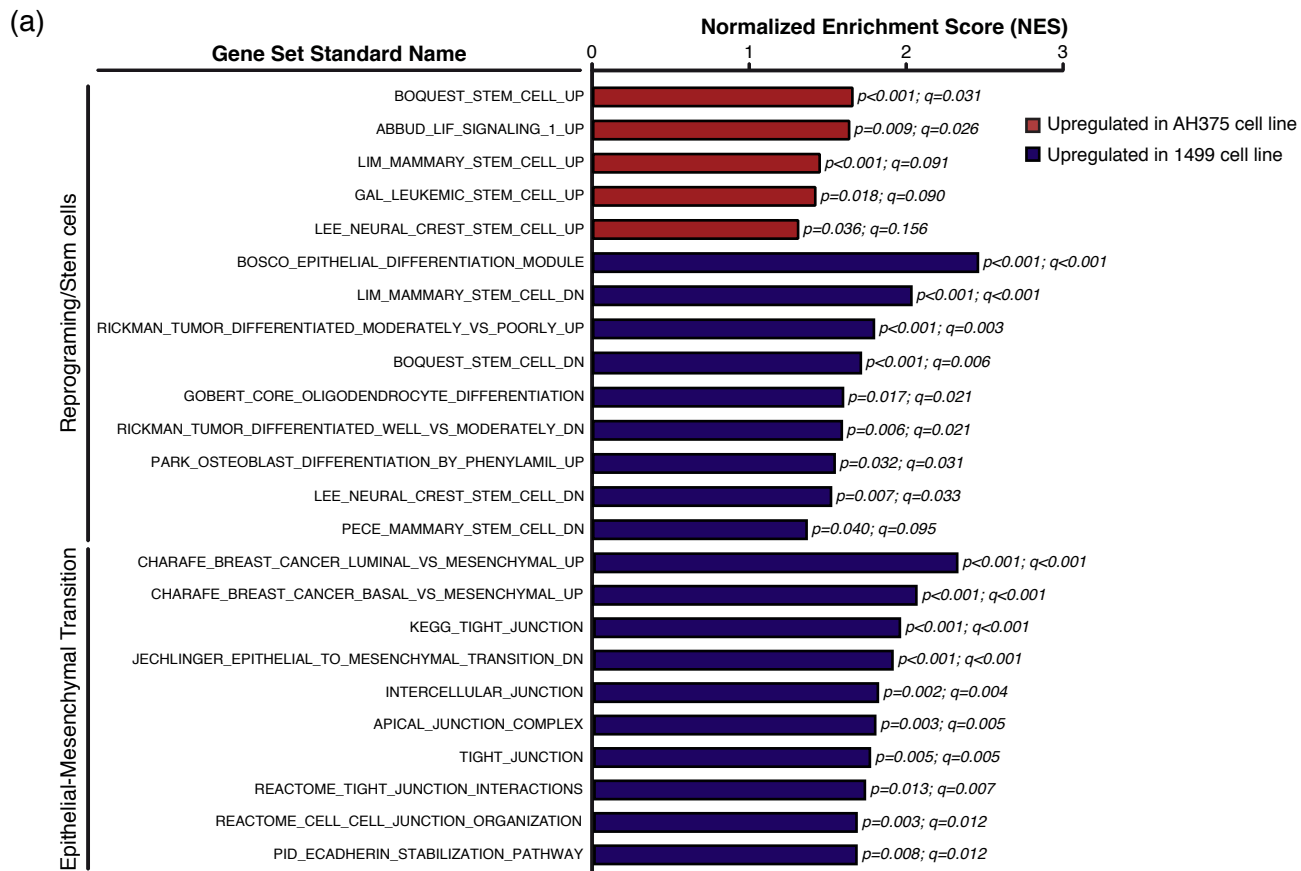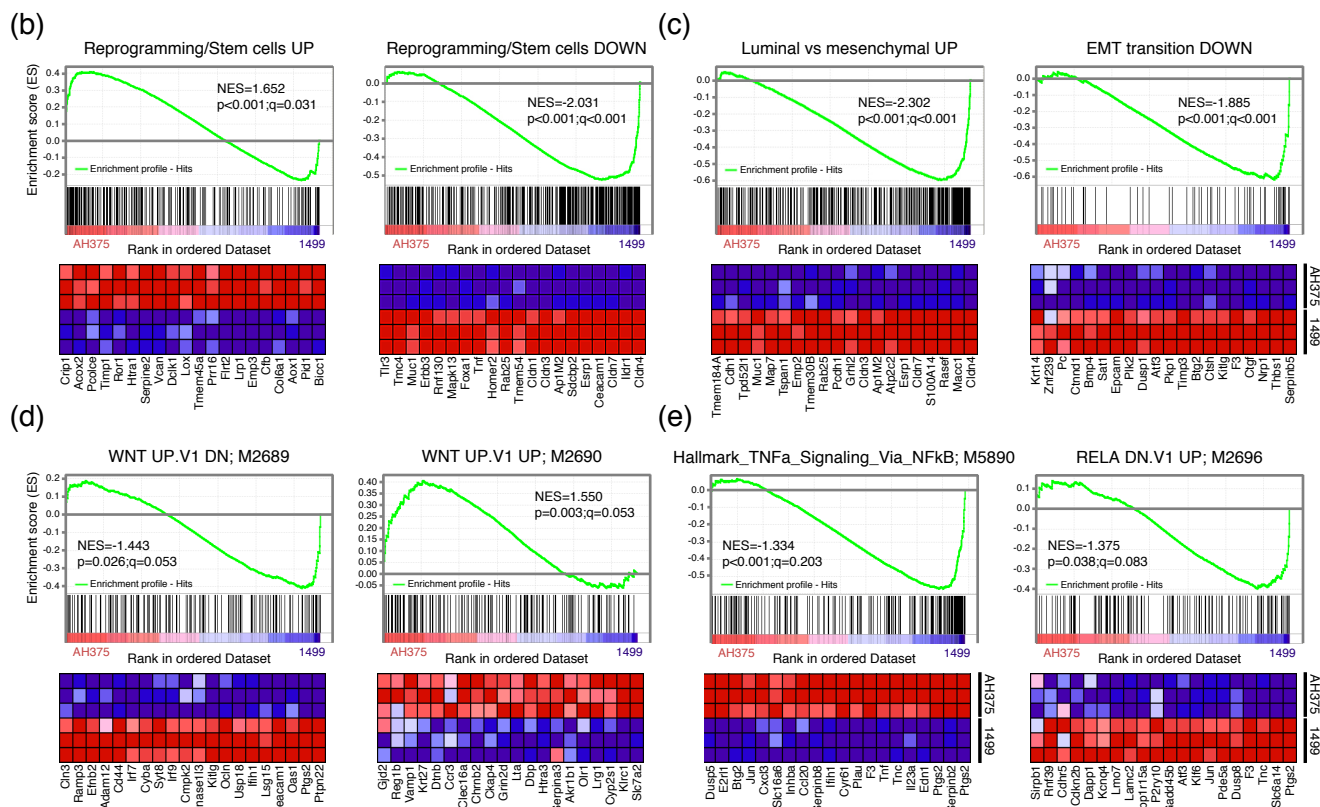

Deschênes-Simard et al. - Fig.S2



(a)

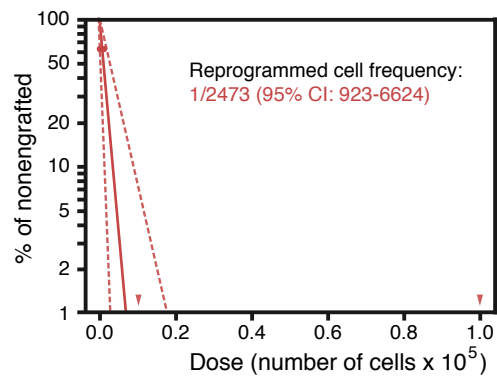

(b)

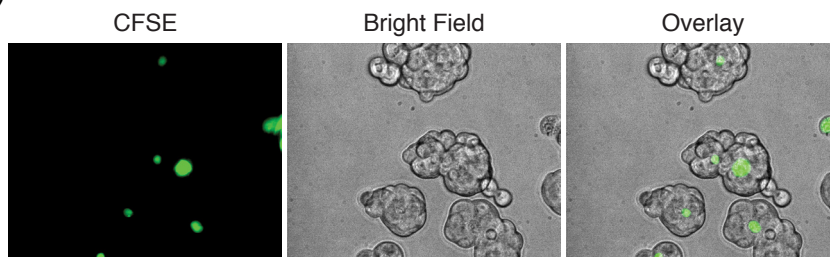

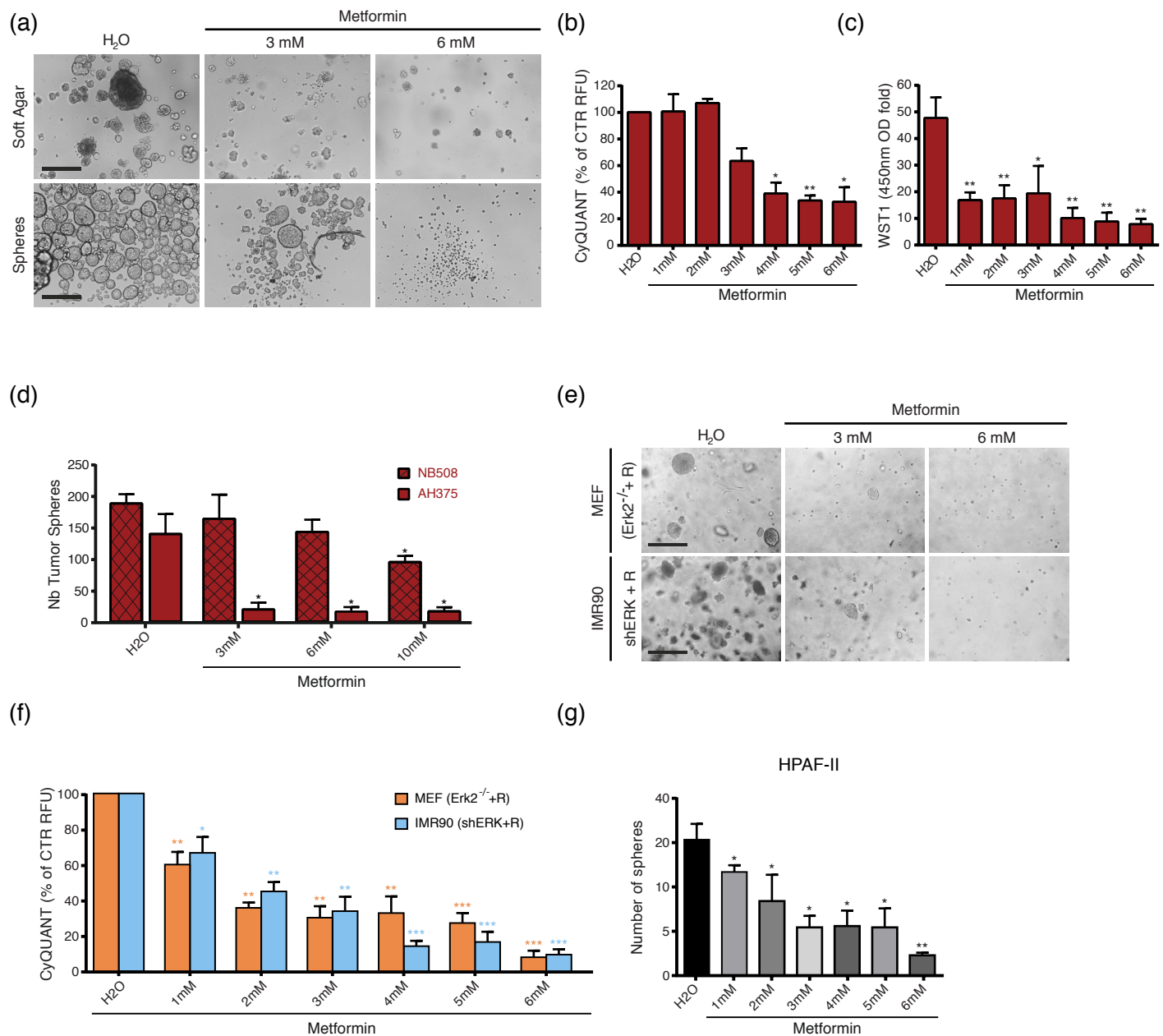

Deschênes-Simard et al. - Fig S5

(a)

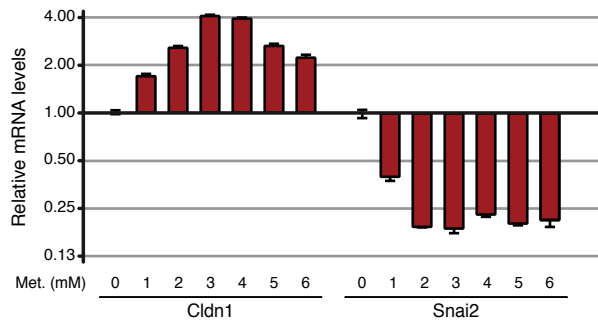

(b)

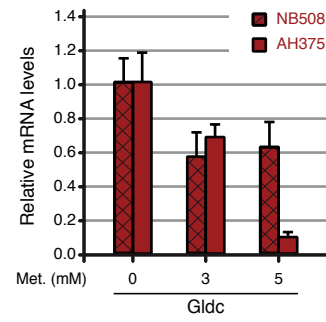

Supplement: Supplementary file 1 [file ACEL-18-e12889-s001.pdf]
