# Supplementary material for: Circumventing senescence is associated with stem cell properties and metformin sensitivity
Source: Aging Cell. 2019 Jan 6;18(2):e12889. doi: 10.1111/acel.12889 (PMC6413657; doi:10.1111/acel.12889)
Supplement: Supplementary file 2 [file ACEL-18-e12889-s002.docx]

**Supplemental Data**

Supplementary Figure S1. Senescence markers of in 1497 PanIN cells in comparison with AH375 PDAC cells. **(a)** Percentage of SA-β-Gal positive cells and SD of triplicates, n=2. HPNE hTERT primary pancreatic epithelial cells were used as negative controls and the same cells treated with camptothecin (25 nM) for four days, were used as positive control. Note that 1497 cells are more senescent that camptothecin treated cells. **(b)** QPCR for Angplt2, Il23a, Il15, Ccl5 and Ccl7 in AH375 (PDAC) and 1497 cells (PanIN). **(c)** QPCR for Il1a as in (c). Note that the Il1a mRNA was not detectable in PDAC cells. **(d)** QPCR for senescence genes in cells as in (c). Error bars indicate mean ± SD, n=3.

Supplementary Figure S2. The transition from PanIN to PDAC involves acquisition of stem cell and EMT gene expression modules. **(a)** Compilation of significant gene expression signatures suggesting stem cell properties and EMT in AH375 cells. Red, gene expression module enriched in AH375 cells; Blue, gene expression module enriched in 1499 cells. (**b**) Gene Set Enrichment Analysis (GSEA) of microarray data revealed several gene expression signatures of stem cells in AH375 and differentiation in 1499 cells; the most significant are shown. (**c**) Numerous gene expression signatures suggest epithelial-mesenchymal transition (EMT) in AH375 cells; the most significant are shown. (**d**-**f**) Gene expression signatures suggesting LIF signaling and WNT pathway activation (WNT UP.V1 DN; M2689) in AH375 cells. (**g**) Genes downregulated by NF-κB (RELA DN.V1 UP; M2696) are enriched in AH375 PDAC cells.

Supplementary Figure S3. Ras-induced transformation of mouse embryonic fibroblasts and human primary cells expressing low ERK levels. (**a**) Volume of solid tumors 32 days after subcutaneous injection into BALB/c nude mice of 1x10^6^ Erk2-null mouse embryonic fibroblasts (MEFs) transduced with an empty vector (V) or a vector expressing HRasG12V (R). (**b**) Volume of cystic tumors 32 days after subcutaneous injection into BALB/c nude mice of 1x10^6^ normal human mammary epithelial cells (HMEC) transduced with an shRNA targeting ERK2 (shERK), a vector expressing hTERT and an empty vector (V) or a vector expressing HRasG12V (R). (**c**) Volume of solid tumors 32 days after subcutaneous injection into BALB/c nude mice of 1x10^6^ normal human fibroblasts from the lung (IMR90) transduced as in (b). (**d**) Representative immunohistochemical staining with anti-p-ERK of tumors as in (c). H&E, hematoxylin and eosin. T, tumor; MS, mouse stroma.

Supplementary Figure S4. Stem cell properties of AH7375 cells. **(a)** Serial limiting dilutions of AH375 cells (10,000, 1,000, 100 cells) were injected subcutaneously in SCID mice. The number of visible tumors for each injection was scored and the data was analyzed with extreme limiting dilution analysis (ELDA) (http://bioinf.wehi.edu.au/software/elda/). We found that 1/2473 cells were capable of initiating tumors. The confidence interval (CI) is indicated. **(b)** Micrograph pictures of tumor spheres of CFSE-stained AH375 cells taken 3 days after CFSE staining.

Supplementary Figure S5. Metformin targets reprogrammed pancreatic cancer cells. (**a**) Metformin inhibits the capacity of AH375 cells to form colonies in soft agar (top) and in tumor sphere assay (bottom). Scale bar = 200μm. (**b**) Quantification of proliferation over a 7-days period in soft agar of cells as in (a) using the CyQuant GR dye. The relative fluorescence unit (RFU) at 520 nm of the control (H_2_O) is expressed as 100% and the other results are expressed as the percent of control. Average of triplicates ± SD, *p < 0.05, **p < 0.01. (**c**) Proliferation of tumor spheres as in (a) over a period of 7 days expressed as the fold of absorbance at 450 nm after coloration with the WST-1 cell proliferation assay. Average of triplicates ± SD, *p < 0.05, **p < 0.01. (**d**) Effects of metformin on reprogrammed pancreatic cancer cells. Metformin inhibits the capacity of AH375 and NB508 cells to form tumor spheres. The number of tumor spheres after 7 days was counted by microscopic examination. Average of triplicates ± SD. (**e**) Metformin inhibits the capacity of HRasG12V (R)-transformed Erk2-null MEF and IMR90 (hTERT+shERK) to form colonies in soft agar. Scale bar = 400μm. (**f**) Quantification of proliferation over a 7-days period in soft agar using the CyQuant GR dye. The relative fluorescence unit (RFU) at 520 nm of the control (H_2_O) is expressed as 100% and the other results are expressed as the percent of control. shERK, shRNA against ERK2; R, HRasG12V. Average of triplicates ± SD, * p < 0.05, ** p < 0.01, *** p < 0.001. **(g)** Number of spheres formed by HPAF-II cells after treatment for 7 days with the indicated concentration of metformin. Average of triplicates ± SD, * p < 0.05, ** p < 0.01.

Supplementary Figure S6. Effects of metformin on gene expression. **(a)** Relative mRNA levels measured by qPCR for Cldn1 (upregulated in epithelial cells) and Snal2 (downregulated in epithelial cells) in AH375 cells treated with the indicated concentration of metformin (Met). Average of triplicates ± SD. (**b**) Metformin inhibits glycine decarboxylase in reprogrammed pancreatic cancer cells. QPCR for glycine decarboxylase (Gldc) in AH375 and NB508 cells treated with the indicated doses of metformin for 5 days. Error bars indicate mean ± SD. * p < 0.05.

**Supplemental Experimental Procedures**

***Reagents and Plasmids***

Metformin hydrochloride (#1396309, Sigma) was diluted in H_2_O and added to cell media at the indicated concentrations. pFG12-CMV-hTERT-IRES-GFP, pWZL-hygro and pWZL-hygro/HRas^G12V^ were described before.(Ferbeyre et al., 2000; Voghel et al., 2010) pLPC-puro/E1A and pLPC-puro/c-MYC-HA is a kind gift from Dr. Scott W. Lowe (Memorial Sloan-Kettering Cancer Center, New-York, NY). The pLKO-puro lentiviral vectors used to express a non-targeting shRNA (shCTR) or a shRNA against ERK2 (shERK) were from Sigma (SHC002, TRCN0000010041).

***Pancreatic Ductal Cell Isolation***

The protocol for isolation of 1497, 1498, 1499 and AH375 cells was adapted from a previously published method.(Schreiber et al., 2004) Briefly, mice were euthanized, the pancreas was gently resected, minced and digested in 10 ml of Hank’s balanced salt solution (HBSS) containing 2 mg/ml collagenase D (#11088858001, Roche Applied Science) during 25 min at 37^o^C with agitation by magnetic stir. The cell suspension was pelleted by centrifugation at 1200 rpm for 5 min and cells were washed once with PBS. Then, cells were resuspended in 3 ml of 0.05% trypsin-EDTA (#25300-054, Gibco) and incubated for 3 min at 37^o^C. Three volumes of DMEM + 10% FBS were added to neutralize trypsin, the cell suspension was filtered through a 85 µM nylon mesh and then pelleted by centrifugation at 1200 rpm for 5 min. Cells were resuspended in a small volume of PBS pH 7.2 + 0.5% BSA + 2 mM EDTA and counted. A suspension of 1 to 1.5 x 10^6^ cells/ml was made in the same buffer and Fluorescein-conjugated DBA (FITC-DBA) lectin (FL-1031, Vector Labs) was added at a dilution 1:400. Cells were incubated at 4^o^C for 10 min and then pelleted by centrifugation at 1200 rpm for 5 min. Cells were washed once with PBS pH 7.2 + 0.5% BSA + 2 mM EDTA and then resuspended in 3 ml of the same buffer. FITC-positive cells were sorted by FACS at low pressure and seeded in wells of a 48-well plate pre-coated with laminin and containing pancreatic medium. The NB508 PDAC cell line was established from the *Pdx1-Cre;LSL-Kras^G12D^;p53^Lox/+^* mouse model as described previously (Bardeesy et al., 2006).

**Cell growth**

All cells were cultured with 5% CO_2_ supply at 37 °C in a humidified incubator. They were cultured in the following media: IMR90, HPAF-II, HEK 293T and MEF in DMEM (#319-015-CL, Wisent) supplemented with 10% fetal bovine serum (FBS; Wisent) and 1% penicillin G/streptomycin sulphate (P/S; Wisent); HMEC in 90% MEGM completed with bovine pituitary extract, human recombinant Epidermal Growth Factor, insulin, hydrocortisone and Gentamicin/Amphotericin as provided in the MEGM Bullet kit (CC-3150; Lonza); NB508 in RPMI (#350-000-CL, Wisent) supplemented with 10% FBS and 1% P/S; 1497, 1498, 1499 and AH375 cells were propagated on surfaces coated with laminin (#354239, BD Biosciences) in pancreatic medium consisting of DMEM-F12 (#11330-032, Gibco) supplemented with 5 mg/ml D-glucose, 1.22 mg/ml Nicotinamide (N-3376, Sigma), 5 nM 3,3’,5-triiodo-L-thyronine (#91990, Sigma), 1 µM Dexamethasone (D1756, Sigma), 100 ng/ml Cholera toxin (C8052, Sigma), 5 ml/L ITS+ (#354352, BD Biosciences), 0.1 mg/ml Soybean Trypsin Inhibitor type 1 (T6522, Sigma), 20 ng/ml EGF (#5331-LF, Cell Signaling Technology), 5% Nu-Serum IV (#355104, BD Biosciences), 25 µg/ml Bovine Pituitary Extract (13028-014, Gibco) and 1% P/S. Adherent cells were trypsinized using trypsine-EDTA 1X (Stock 10X; 325-052-EL, Wisent) except for mouse pancreatic ductal cells. For the latter, cell dissociation was done using TrypleE (12604-013, Gibco).

***Cell Proliferation Assays and SA-β-Gal***

Cells were plated in 48-well plates at 5000 cells per well in 0.5 ml of media. At the indicated time points, 50 µl of 12 mM MTT reagent (M-6494, Molecular Probes) was added to each well and cells were incubated 3 hours at 37^o^C. After incubation, the medium was aspirated, and the cells dried at room temperature. Then, the formazan was solubilised with 200µl DMSO and the absorbance at 540 nm was measured using a microplate reader. The relative proliferation is expressed as the fold of absorbance between a specific time point and the beginning of the experiment (day 0). SA-β-Gal was performed as previously described (Deschenes-Simard et al., 2013).

***Soft Agar Assays***

Soft agar assays with MEF and IMR90 cells were done on 96-well flat-bottom microplate with 5000 cells seeded per well by using the CytoSelect^TM^ 96-Well Cell Transformation Assay kit (CBA-130, Cell Biolabs) as described previously.(Deschenes-Simard et al., 2013) For mouse pancreatic cells, the protocol was adapted to substitute the required specific culture medium. Agar was directly solubilized in the culture medium by incubating at 95^o^C to obtain a concentrated 2.4% agar solution. This solution was cooled to 37^o^C in a water bath and then medium was added to obtain a final solution of 0.6% agar. The latter solution was used for the base agar layer and to mix with cell suspensions. The normal protocol was then followed, but with the specific medium.

***Sphere-Forming Assays***

To examine the capacity of tumor sphere formation, cells were trypsinized and washed with PBS to remove FBS. Cells were cultured in ultra-low attachment 24-well plates (#3473, Corning) (1000 cells/well) and in 1 ml of CSC medium, consisting of DMEM-F12 (#11039-021, Gibco) supplemented with B-27 Supplement (#17504-044, Gibco), 20 ng/ml EGF (mouse cells: #5331-LF, human cells: #8916-LF, Cell Signaling Technology) and 20 ng/ml βFGF (mouse cells: #5414-LF, human cells: #8910-LF, Cell Signaling Technology). The cells were incubated in a humidified atmosphere with 5% CO_2_ at 37^o^C for the indicated period of time and supplements (B-27, EGF, βFGF) were added every 4 days at the above concentration. Spheroids were counted using a microscope. The sphere size was evaluated by an automated analysis of pictures with the software CellProfiler (Broad Institute). To evaluate cell proliferation two methods were used: 1) cells (one day after seeding) and spheroids (at the experiment end point) were incubated with 100 µl of premixed WST-1 reagent (#630118, Clontech) for 3 hours at 37^o^C. The absorbance at 450 nm was measured using a microplate reader and the results are expressed as the fold of absorbance. 2) Spheroids were manually counted during microscopic examination. For CFSE staining of tumor spheres, AH375 cells were stained with CellTrace™ CFSE Cell Proliferation Kit (Thermofisher #C34554) following manual instructions. Stained cells were then seeded in suspension in tumor sphere medium at 1000 cells/well in 12 well plates.

***Immunoblotting***

Adherent cells were washed with PBS and then scraped on ice into 250 to 500 µl of ice cold RIPA buffer (50 mM Tris-Hcl pH8.0, 150 mM NaCl, 1% Triton X-100, 0.1% SDS, 0.5% Na-deoxycholate, 1 mM EDTA, 1 mM EGTA) containing 1X Complete-EDTA free Protease Inhibitor Cocktail (#11 873 580 001, Roche Applied Science) and 1X PhosSTOP Phosphatase Inhibitor Cocktail (#04 906 837 001, Roche Applied Science). Cell lysates were kept on ice for 15 min, sonicated 40 seconds at a low intensity and then cleared by centrifugation at 13 000 RPM for 20 min. Protein concentration was evaluated using the Micro BCA Protein Assay Kit (#23235, Thermo Scientific) and the concentration of samples was normalized by adding appropriated quantity of RIPA buffer. The samples were completed with 1X Laemmli buffer (Stock of 6X: 12% SDS, 47% glycerol, 60 mM Tris pH6.8, 0.06% bromophenol blue) and 10% β-mercaptoethanol. Then the lysates were boiled 5 min and conserved at -80^o^C until migration. Proteins were separated by SDS-PAGE and to Immobilon-P PVDF membrane (IPVH00010, Millipore). Membranes were blocked 1 hour at room temperature in Tris-buffered saline containing 0.1% Tween 20 (TBS-T) and 5% dry milk and then washed 3 times 5 min with TBS-T. The membranes were incubated with the primary antibodies diluted in TBS-T + 3% BSA + 0.05% Na-azide overnight at 4^o^C or 1 hour at room temperature. The following primary antibodies were used: anti-c-MYC (1:1000; clone D84C12, #5605, Cell Signaling Technology), anti-ERK1/2 (1:400; Sc-94 (K-23), Santa Cruz Biotechnology), anti-phospho-ERK1/2^T202/Y204^ (1:2000; clone D13.14.4E, #4370, Cell Signaling Technology), anti-H-Ras (1:250; clone F235, Sc-29, Santa Cruz Biotechnology), anti-IκBα (1:1000; #9242, Cell Signaling Technology), anti-phospho-IκBα^S32^ (1:1000; clone 14D4, #2859, Cell Signaling Technology), anti-Nanog (1:2000; clone D73G4, #4903, Cell Signaling Technology), anti-STAT3 (1:1000; clone 124H6, #9139, Cell Signaling Technology), anti-phospho-STAT3^S727^ (1:1000; #9134, Cell Signaling Technology), anti-phospho-STAT3^Y705^ (1:1000; #9131, Cell Signaling Technology), anti-α-Tubulin (1:20000; clone B-5-1-2, T6074, Sigma-Aldrich), anti-β-actin (1:10000; clone 8H10D10, #3700, Cell Signaling Technology). Membranes were washed 3 times 5 min with TBS-T and then incubated with the secondary antibodies diluted in TBS-T + 5% dry milk 1 hour at room temperature. The following secondary antibodies were used: goat anti-rabbit IgG conjugated to HRP (1:3000, #170-6515, Bio-Rad) or goat anti-mouse IgG conjugated to HRP (1:3000, #170-6516, Bio-Rad). Finally, the membranes were washed 3 times 5 min with TBS-T. Immunoblots were visualised using enhanced chemiluminescence (ECL) detection systems and Super RX X-Ray films (Fujifilm) or a ChemiDoc^TM^ MP system (Bio-Rad). Band quantification was done using ImageJ or Image Lab 4.0 (Bio-Rad).

***Immunofluorescence***

For paraffin-embedded tissues, slides with tissue samples were deparaffinized and rehydrated by sequential incubation in xylenes (3 x 5 min), 100% ethanol (2 x 2 min), 95% ethanol (1 x 2 min), 70% ethanol (1 x 2 min) and dH_2_O (2 x 2 min). Heat-induced epitope retrieval was performed using a pressure cooker for 20 min in Tris-EDTA-based buffer pH 9.0 (ab93684, Abcam). Slides were cooled down for at least 1 hour, washed once for 5 min with PBS and once for 5 min with PBS + Tween 20 (PBS-T). Tissues were delimited using a hydrophobic barrier pen. Tissues were blocked 1 hour at room temperature with PBS-T + 2% BSA and then incubated with anti-c-MYC (1:800; clone D84C12, #5605, Cell Signaling Technology) or anti-phospho-ERK1/2^T202/Y204^ (1:400; clone E10, #9106, Cell Signaling Technology) diluted in the same buffer overnight at 4^o^C. Slides were rinsed 3 times for 3 min in PBS-T and then incubated with donkey anti-rabbit AlexaFluor 488 (1:400; A-21206, Molecular Probes) diluted in PBS-T + 2% BSA for 45 min at room temperature. Slides were washed 3 times for 5 min in PBS-T and then mounted with Vectashield Mounting Medium with DAPI (H-1200, Vector Labs). Images were captured with a Nikon Eclipse 80i upright microscope.

For cell lines 1498 (passage 7) or AH375 cells (passage 21) were seeded on mouse laminin I coated coverslips (NeuVitro). Cells were fixed on coverslips with paraformaldehyde 4%, then permeabilized in a solution of PBS1X, 2% BSA, 0,2%Triton X-100. Cells were blocked with a solution of PBS1X, 2%BSA and immunostained for 1 hour at 4°C with the following primary antibodies: anti-53BP1 rabbit polyclonal (1:200, Ab-1, Cat# PC712, lot: D00137736, Calbiochem, EMD Biosciences, San Diego, CA); anti-phospho-γH2A.XS139 mouse monoclonal (1:175, JBW-301, lot: 2552645, Millipore, Billerica, MA); anti-HP1γ mouse monoclonal (1:400, MAB3450, lot JC1686017, Millipore Billerica, MA). Appropriate secondary antibodies were used for immunostaining during 1h at RT in the dark: goat anti-mouse IgG AlexaFluor 488 (1:1000; A11029, Lot 1423008, Molecular Probes, Eugene, OR); goat anti-rabbit IgG AlexaFluor 568 (1:1000, A11036, Lot 1504529, Molecular Probes, Eugene, OR). Coverslips were mounted with ProLong Gold antifade reagent with DAPI (P36935, Molecular Probes, Eugene, OR). Images were acquired with a FV300 Olympus confocal microscope with a PMT 1st generation and Fluoview V4.2. Images were processed with ImageJ.

***Immunohistochemical Analyses***

Tissue samples were melted at 55^o^C for 1 hour and then deparaffinized and rehydrated by sequential incubation in xylenes (2 x 5 min), 100% ethanol (1 x 5 min), 95% ethanol (1 x 3 min), 75% ethanol (1 x 3 min) and 40% ethanol (1 x 3 min). Samples were washed in PBX/0.3% Triton X-100 5 min and then in dH_2_O 3 min. Heat-induced epitope retrieval was performed using a pressure cooker for 20 min in citrate-based buffer (H-3300, Vector Labs). Samples were cooled down for at least 1 hour and then washed 3 times during 3 min with PBS/0.3% Triton X-100. Endogenous peroxidase was inactivated by incubation for 10 min at room temperature in a solution of 1% H_2_O_2_. Samples were washed 3 times for 3 min in PBS/0.3% Triton X-100 and tissues were delimited using a hydrophobic barrier pen. Tissues were blocked 1 hour at room temperature with 5% goat serum in PBS/0.3% Triton X-100, and then incubated with primary antibodies diluted in the same buffer overnight at 4^o^C. The following primary antibody was used: anti-phospho-ERK1/2^T202/Y204^ (1:400; clone D13.14.4E, #4370, Cell Signaling Technology). Tissues were washed 3 times for 3 min in PBS/0.3% Triton X-100 and then incubated with secondary antibodies diluted in PBS/0.3% Triton X-100 + 5% goat serum during 1 hour at room temperature. The following secondary antibody was used: biotinylated goat anti-rabbit IgG (1:200; BA-1000, Vector Labs). Slides were incubated with ABC Elite reagent (PK-6100, Vector Labs) 30 min at room temperature (prepared 30 min before incubation), and then washed 3 times for 3 min with PBS/0.3% Triton X-100. Finally, the specimens were stained for peroxidase with Di-amine-benzidine (DAB) substrate kit (SK-4100, Vector Labs). The reaction was stopped by washing with water when the staining was sufficient; the same incubation time was applied to all samples. Tissues were counterstained with hematoxylin ad eosin (H9627, Sigma) and dehydrated by sequential incubation in 40% ethanol (1 x 1 min), 75% ethanol (1 x 1 min), 95% ethanol (1 x 1 min), 100% ethanol (1 x 1 min) and xylenes (2 x 5 min). Finally, slides were mounted with Sub-X Mounting Medium (#13519, EMS).

***Microarrays***

*RNA quality, cDNA synthesis and data acquisition*

Total RNA extracts were prepared in TRIzol (#15596-026, Ambion), and their purification was performed according to the manufacturer’s instructions. The RNA solution was further purified using the RNeasy MinElute Cleanup Kit (#74204, Qiagen). Solutions of 100 ng/µl RNA were sent to Génome Québec Innovation Centre for RNA processing, hybridation on GeneChip® Human Gene 1.0 ST Array or GeneChip® Mouse Gene 2.0 ST Array (Affymetrix) and data acquisition. The microarray data discussed were deposited in NCBI’s Gene Expression Omnibus (GEO) and are accessible through GEO Series accession numbers GSE33613 and GSE57566.

The Eukaryote Total RNA Nano assay of the Agilent 2100 Bioanalyzer (Agilent Technologies) was used to validate total RNA integrity. The protocol from the Ambion® WT Expression kit for Affymettrix® GeneChip®Whole Transcript (WT) Expression Arrays (Applied Biosystems) was followed to prepare cDNAs. The single-stranded cDNAs were treated with Uracil DNA glycosylase, fragmented with APE1 enzyme and labelled with Biotin Allonamide Triphosphate according to the instructions of the GeneChip® WT Terminal Labeling Kit (Applied Biosystems). Labelled cDNAs were hybridized for 16 hours at 45^o^C with rotation at 60 rpm on the appropriated DNA Gene Chip. The DNA chips were washed and stained in the Affymetrix Fluidics Station 400 according to the manufacturer’s instructions and then scanned on a GeneChip® Scanner 3000 7G/4 Color Early Access (Affymetrix).

*Data normalization and analysis*

Data from triplicates of each condition were analyzed with the FlexArray 1.6.3 software (Blazejczyk, Miron, & Nadon, 2007) using Affymetrix default analysis settings. Raw data were normalized with a Robust Multi-array Average (RMA) algorithm, the mean fold change in the signal intensity between conditions was determined for all transcripts and a two-sample student's t-test was used to identify significant changes. Transcripts with a fold changes greater than ± 2 and a p-value ˂0.05 were considered for further analysis.

*Bioinformatics analyses*

*Inferring biological functions*

A FatiGO single enrichment analysis with the Babelomics 4.3 platform (Medina et al., 2010) was performed to infer biological functions affected between experimental conditions. The Gene Ontology (GO) and Kyoto Encyclopedia of Genes and Genomes (KEGG) terms significantly enriched among transcripts when compared with the rest of the genome were identified. These terms and their associated transcripts were grouped in general categories as indicated.

*Prediction of regulated transcription factors*

The Distant Regulatory Elements of Co-regulated genes (DiRE) web-based application (Gotea & Ovcharenko, 2008) was used to predict regulated transcription factors (TF). A random set of 5000 genes was used as the source of background genes.

*Gene set enrichment analysis*

An unbiased global analysis of gene set enrichment with The Gene set Enrichment Analysis (GSEA) 2.0.14 software (Subramanian et al., 2005) was used to identify the relevant phenotypes significantly over represented in the experimental conditions of interest. A positive NES indicates a correlation with the phenotype that is compared to the phenotype used as a reference, while a negative NES indicates correlation with the opposite phenotype (the reference). The significance of NES values is determined by the False Discovery Rate (FDR; q-value) and by a nominal p-value calculated by using an empirical phenotype-based permutation test procedure. The threshold of significance was set at a p-value ≤ 0.05 and a q-value ≤ 0.25. The ranking metric used was the signal-to-noise ratio and 1000 permutations were performed.

*Database for Annotation, Visualization and Integrated Discovery (DAVID)*

DAVID v6.7 (Huang da, Sherman, & Lempicki, 2009a, 2009b) was used for Figure 5a and 5b. An unbiased analysis of the whole microarray dataset was used to retrieve significant functional gene clusters, as well as the GO and KEGG terms enriched among these clusters.

***Quantitative PCR (qPCR)***

Total mRNA extracts were prepared in TRIzol (Invitrogen) as described before.(Vernier et al., 2011) Gene expression levels were determined with a lightCycler 480 Real-Time PCR System (Roche Applied Science) according to the SYBR Green technology and the relative quantification was determined by using the ΔΔCT method. The primers used are presented below:

Table SI. List of primers used for qPCR

| **Target** | **Forward primer (5’→3’)** | **Reverse primer(5’→3’)** |
| --- | --- | --- |
| **Actb*** | TCCTAGCACCATGAAGATCAAGATC | CTGCTTGCTGATCCACATCTG |
| **Angptl2** | CCCTGGAGGTTGGACTGTCATC | CGATGTTCCCAAACCCTTGCTT |
| **C2cd5** | GGTAAAGGTTGTCTTATTCAGGCAAGG | GGCAAGAGATTACTGATAGCTGTGG |
| **Ccl5** | CCCCTACTCCCACTCGGTCCT | TGATTTCTTGGGTTTGCTGTGC |
| **Ccl7** | ATGAGGATCTCTGCCACGCTTC | TAGCAGCATGTGGATGCATTGG |
| **Cdkn1a** | TCCACAGCGATATCCAGACA | GGACATCACCAGGATTGGAC |
| **Cldn1** | CGACTCCTTGCTGAATCTGAACAGT | TGGACACAAAGATTGCGATCAG |
| **Fam214b** | CCCAAGGAGCCTGTTTTGGA | TCGAAGGGAGCTTAGCTTCAGG |
| **Gcsh** | CACCGGATCTGCTTTGCTGTC | GCTGATTCCCACCGTTCCAATA |
| **Gldc** | TCAATAGCTCCTCCGAACTTGC | GCACAAAGGGGTGGATGTTGG |
| **Glul** | ctcgctctcctgacctgttc | ttcaagtgggaacttgctga |
| **Igfbp4** | CATTCCAAACTGTGACCGCAAC | CTGTCTTCCGATCCACACACCA |
| **Il15** | CAGAGGCCAACTGGATAGATGT | TGCAACTGGGATGAAAGTCACTG |
| **Il1a** | TCCATAACCCATGATCTGGAAGA | TTTGGTTGAGGGAATCATTCAT |
| **Il23a** | TCCCTACTAGGACTCAGCCAACTC | GAACTCAGGCTGGGCATCTGTT |
| **Plk3** | CCCCAGAGCAGAGGAAAAAGAC | ATGGCCCTGTCTCAGCAGCA |
| **Plxna3** | TGTCACCAAGTACCGTCAGGAGA | GCTGGACACCAGGGTGATGAT |
| **Shmt2** | TGACCCTGAGATGTGGGAGCTT | AGCTCGGCTGCAGAAGTTCTCT |
| **Snai2** | TGTGGCAAGGCTTTCTCCAG | AGAAAGGCTTTTCCCCAGTGT |
| **Sparc** | AATTTGAGGACGGTGCAGAGGA | CATGTTTGCAATGATGGTTCTGG |
| **Stat3**  **Tbp*** | GCCTGTCTGCAGAGTTCAAGCA  GTTTCTGCGGTCGCGTCATTTT | GCAGCTCCTCAGTCACGATCAA  TCTGGGTTATCTTCACACACCATGA |
| **Tmem87b** | CCTGTCACAATGATTACCCTGATCT | CAGACATCTGGATCAGCATGAAGT |
| **Trdmt1** | GGTTGCGAGAGGATGGAACC | TGTGCAGGGATATGACTTTCTCG |
| **Tspan13** | TCCTCGTGCGCCCCCATAAT | GTCAGCCAAACACCCAGGATCT |
| **Vim** | aaagcgtggctgccaagaac | cagcctcagagaggtcagcaaa |

*: housekeeping genes

**Supplemental References**

Bardeesy, N., Aguirre, A. J., Chu, G. C., Cheng, K. H., Lopez, L. V., Hezel, A. F., Depinho, R. A. (2006). Both p16(Ink4a) and the p19(Arf)-p53 pathway constrain progression of pancreatic adenocarcinoma in the mouse. *Proc Natl Acad Sci U S A, 103*(15), 5947-5952. doi:10.1073/pnas.0601273103

Blazejczyk, M., Miron, M., & Nadon, R. (2007). FlexArray: A statistical data analysis software for gene expression microarrays. *Genome Quebec, Montreal, Canada,* [*http://genomequebec.mcgill.ca/FlexArray*](http://genomequebec.mcgill.ca/FlexArray).

Deschenes-Simard, X., Gaumont-Leclerc, M. F., Bourdeau, V., Lessard, F., Moiseeva, O., Forest, V., Ferbeyre, G. (2013). Tumor suppressor activity of the ERK/MAPK pathway by promoting selective protein degradation. *Genes Dev, 27*(8), 900-915. doi:gad.203984.112 [pii] 10.1101/gad.203984.112

Ferbeyre, G., de Stanchina, E., Querido, E., Baptiste, N., Prives, C., & Lowe, S. W. (2000). PML is induced by oncogenic ras and promotes premature senescence. *Genes Dev, 14*(16), 2015-2027.

Gotea, V., & Ovcharenko, I. (2008). DiRE: identifying distant regulatory elements of co-expressed genes. *Nucleic Acids Res, 36*(Web Server issue), W133-139. doi:gkn300 [pii] 10.1093/nar/gkn300

Huang da, W., Sherman, B. T., & Lempicki, R. A. (2009a). Bioinformatics enrichment tools: paths toward the comprehensive functional analysis of large gene lists. *Nucleic Acids Res, 37*(1), 1-13. doi:10.1093/nar/gkn923

Huang da, W., Sherman, B. T., & Lempicki, R. A. (2009b). Systematic and integrative analysis of large gene lists using DAVID bioinformatics resources. *Nat Protoc, 4*(1), 44-57. doi:nprot.2008.211 [pii] 10.1038/nprot.2008.211

Medina, I., Carbonell, J., Pulido, L., Madeira, S. C., Goetz, S., Conesa, A., Dopazo, J. (2010). Babelomics: an integrative platform for the analysis of transcriptomics, proteomics and genomic data with advanced functional profiling. *Nucleic Acids Res, 38* (Web Server issue), W210-213. doi:gkq388 [pii]10.1093/nar/gkq388

Schreiber, F. S., Deramaudt, T. B., Brunner, T. B., Boretti, M. I., Gooch, K. J., Stoffers, D. A., Rustgi, A. K. (2004). Successful growth and characterization of mouse pancreatic ductal cells: functional properties of the Ki-RAS(G12V) oncogene. *Gastroenterology, 127*(1), 250-260.

Subramanian, A., Tamayo, P., Mootha, V. K., Mukherjee, S., Ebert, B. L., Gillette, M. A., . . . Mesirov, J. P. (2005). Gene set enrichment analysis: a knowledge-based approach for interpreting genome-wide expression profiles. *Proc Natl Acad Sci U S A, 102*(43), 15545-15550. doi:10.1073/pnas.0506580102

Vernier, M., Bourdeau, V., Gaumont-Leclerc, M. F., Moiseeva, O., Begin, V., Saad, F., Ferbeyre, G. (2011). Regulation of E2Fs and senescence by PML nuclear bodies. *Genes Dev, 25*(1), 41-50. doi:25/1/41 [pii]10.1101/gad.1975111

Voghel, G., Thorin-Trescases, N., Mamarbachi, A. M., Villeneuve, L., Mallette, F. A., Ferbeyre, G., Thorin, E. (2010). Endogenous oxidative stress prevents telomerase-dependent immortalization of human endothelial cells. *Mech Ageing Dev, 131*(5), 354-363. doi:10.1016/j.mad.2010.04.004
